
JOURNAL INFORMATION
==============================
NLM Title Abbreviation: Hum Brain Mapp
Journal ID: Hum Brain Mapp
NLM Journal ID: 9419065
Journal ID: HBM

Human Brain Mapping

ISSN: 1065-9471
EISSN: 1097-0193
Publisher: Wiley

ARTICLE INFORMATION
==============================
PMCID: PMC11474615
DOI: 10.1002/hbm.25932
Article ID: HBM25932
Article version: 1
Subjects: Issue Information, Issue Information

Editorial board ‐ TOC

Electronic publication date: 2023 Aug 3
Publication date: 2023 Sep
Volume: 44
Issue: 13
First page: 4635
Last page: 4636
Copyright: © 2023 The Authors. Human Brain Mapping published by Wiley Periodicals LLC.
License: This is an open access article under the terms of the http://creativecommons.org/licenses/by/4.0/ License, which permits use, distribution and reproduction in any medium, provided the original work is properly cited.
License URL: https://creativecommons.org/licenses/by/4.0/

Figure count: 0
Table count: 0
Page count: 7

source-schema-version-number: 2.0
cover-date: September 2023
details-of-publishers-convertor: Converter:WILEY_ML3GV2_TO_JATSPMC version:6.4.9 mode:remove_FC converted:15.10.2024

==============================

### HUMAN BRAIN MAPPING

Open Access

Volume 44, Issue 13, September, 2023
View this journal online at wileyonlinelibrary.com

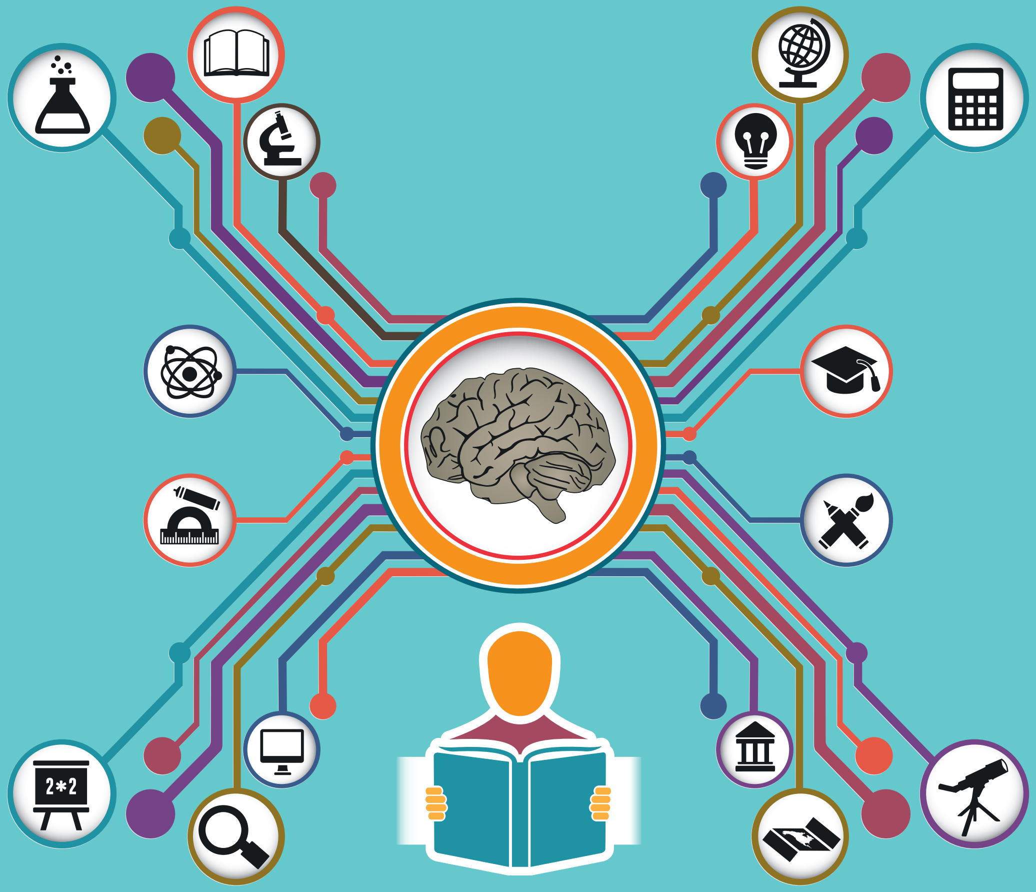

WILEY

ISSN 1097-0193

### HUMAN BRAIN MAPPING

### A Journal Devoted to Functional Neuroanatomy and Neuroimaging

### *Editor-in-Chief*

**Simon B. Eickhoff**
Institute for Systems Neuroscience
Heinrich-Heine University, Düsseldorf, Germany

### *Deputy Editors-in-Chief*

**Gary Egan**
Monash Biomedical Imaging
Monash University, Victoria, Australia

**Sophia Frangou**
Icahn School of Medicine
at Mount Sinai, New York, USA

**Martijn van den Heuvel**
Brain Center Rudolf Magnus,
University of Medical Center,
Utrecht, Netherlands

### *Associate Editors*

**Annabel Chen**
Nanyang Technological University, Singapore

**Michael J. Farrell**
Monash University, Victoria, Australia

**Massimo Filippi**
Università Vita-Salute San Raffaele, Milan,
Italy

**Sophia Frangou**
Icahn School of Medicine at Mount Sinai
New York, USA

**Cheryl L. Grady**
Rotman Research Institute
Toronto, Canada

**Joachim Gross**
Centre for Cognitive Neuroimaging
University of Glasgow, Glasgow, UK

**Martin A. Lindquist**
John Hopkins, Bloomberg School of Public
Health, MD, USA

**Tomáš Paus**
Rotman Research Institute
Toronto, Canada

**Indira Tendolkar**
Radboud University, Nijmegen,
The Netherlands

**Paul Thompson**
Laboratory of Neuro Imaging,
University of Southern California,
Los Angeles, CA, USA

**Helen Zhou**
Yong Loo Lin School of Medicine, National
University of Singapore, Singapore

### *Editorial Board*

**Susan Bookheimer**
Center for Cognitive Neuroscience
UCLA School of Medicine, USA

**Allen R. Braun**
NIDCD, National Institutes of Health
Bethesda, USA

**Christian Buechel**
Univ-Krankenhaus Eppendorf
Hamburg, Germany

**Marco Catani**
King's College London, UK

**Franco Cauda**
University of Turin, Italy

**Vincent Clark**
University of New Mexico, USA

**Olivier David**
Université Joseph Fourier, France

**Mark D'Esposito**
University of California, Berkeley,
USA

**Julien Doyon**
Functional Neuroimaging Unit
University of Montreal, Canada

**Eugene Duff**
University of Oxford, UK

**Timothy Q. Duong**
Research Imaging Institute,
University of Texas Health Science
Center, USA

**Paul Fletcher**
University of Cambridge, UK

**Jia-Hong Gao**
Brain Research Imaging
Center The University of Chicago,
USA

**David C. Glahn**
Yale University School of Medicine
Hartford, USA

**Gregory S. Hickok**
Center for Cognitive
Neuroscience University of California,
Irvine, USA

**Barry Horwitz**
NIDCD, National Institutes of Health
USA

**Peter Jezzard**
John Radcliffe Hospital
University of Oxford, UK

**David N. Kennedy**
Harvard Medical School, USA

**Neda Ladbon-Bernasconi**
Montreal Neurological Institute
Canada

**Nicholas Lange**
Harvard Medical School, USA

**Tatia M. C. Lee**
State Key Laboratory of Brain &
Cognitive Sciences, The University of
Hong Kong

**Louis Lemieux**
UCL Institute of Neurology, UK

**Bernard Mazoyer**
CNRS/CEA, Université de Bordeaux
France

**Gregory McCarthy**
Yale University, USA

**A. Randy McIntosh**
Rotman Research Institute
Toronto, Canada

**Janine Mendola**
McGill Vision Research
McGill University, Canada

**Thomas Nichols**
University of Warwick, UK

**William Penny**
Wellcome Trust Centre for
Neuroimaging, UCL Institute of
Neurology, UK

**Laurent Petit**
CNRS, Université de Bordeaux,
France

**Russel A. Poldrack**
Stanford Unversity, USA

**Jean-Baptiste Poline**
Imagen Orsay Service Hospitalier
Frédéric Joliot, France

**Stephen Smith**
Center for Functional MRI of the
Brain, University of Oxford, UK

**Elizabeth Sowell**
Children'sHospital Los Angeles, USA

**Li Hai Tan**
Shenzhen Institute of Neuroscience
Shenzhen, P.R. China

**Kate Watkins**
John Radcliffe Hospital
University of Oxford, UK

**Richard G. Wise**
Cardiff University, UK

**Roger Woods**
UCLA School of Medicine, USA

**Mark Woolrich**
Oxford Centre for Human Brain
Activity, University of Oxford, UK

**Jinhu Xiong**
University of Iowa, USA

**Robert Zatorre**
MontrealNeurological Institute, Canada

**Open Access and Copyright**
All articles published by *Human Brain Mapping* submitted after January 1, 2020 are fully open access: immediately freely available to read, download, and share. All articles accepted from August 6, 2019 are published under the terms of a Creative Commons license.
Copyright on any research article published by *Human Brain Mapping* is retained by the author(s).

Further information about open access licenses and copyright can be found at: https://authorservices.wiley.com/open-research/open-access/index.html. Information on the licenses used by *Human Brain Mapping* can be found at: https://onlinelibrary.wiley.com/journal/10970193.

**Disclaimer**
The Publisher and Editors cannot be held responsible for errors or any consequences arising from the use of information contained in this journal; the views and opinions expressed do not necessarily reflect those of the Publisher and Editors, neither does the publication of advertisements constitute any endorsement by the Publisher and Editors of the products advertised. Wiley Open Access articles posted to repositories or websites are without warranty from Wiley of any kind, either express or implied, including, but not limited to, warranties of merchantability, fitness for a particular purpose, or non-infringement. To the fullest extent permitted by law Wiley disclaims all liability for any loss or damage arising out of, or in connection, with the use of or inability to use the content.

A Journal Devoted to
Functional Neuroanatomy
and Neuroimaging

### HUMAN BRAIN MAPPING

VOLUME 44, NUMBER 13, SEPTEMBER, 2023

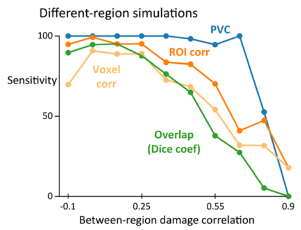

*See article by, Magnotti et al., pages 4738-4753, this issue*

### CONTENTS

◆ *4637* The robustness of persistent homology of brain networks to data acquisition-related non-neural variability in resting state fMRI
**Sidharth Kumar, Ahmedur Rahman Shovon, and Gopikrishna Deshpande**
***Published online 14 July 2023***

◆ *4652* Brain volumes in alcohol use disorder: Do females and males differ? A whole-brain magnetic resonance imaging mega-analysis
**Eleonora Maggioni, Maria G. Rossetti, Nicholas B. Allen, Albert Batalla, Marcella Bellani, Yann Chye, Janna Cousijn, Anna E. Goudriaan, Robert Hester, Kent Hutchison, Chiang-Shan R. Li, Rocio Martin-Santos, Reza Momenan, Rajita Sinha, Lianne Schmaal, Nadia Solowij, Chao Suo, Ruth J. van Holst, Dick J. Veltman, Murat Yücel, Paul M. Thompson, Patricia Conrod, Scott Mackey, Hugh Garavan, Paolo Brambilla, and Valentina Lorenzetti**
***Published online 12 July 2023***

◆ *4667* Differential effects of expectancy on memory formation in young and older adults
**Tineke K. Steiger, Mushfa Yousuf, and Nico Bunzeck**
***Published online 27 June 2023***

◆ *4679* Resting state network connectivity alterations in HIV: Parallels with aging
**Brandon J. Lew, Marie C. McCusker, Jennifer O'Neill, Sara H. Bares, Tony W. Wilson, and Gaelle E. Doucet**
***Published online 7 July 2023***

◆ *4692* Spatiotemporal profile of atrophy in the first year following moderate-severe traumatic brain injury
**Daniel J. Brennan, Jeffrey Duda, Jeffrey B. Ware, John Whyte, Joon Yul Choi, James Gugger, Kristen Focht, Alexa E. Walter, Tamara Bushnik, James C. Gee, Ramon Diaz-Arrastia, and Junghoon J. Kim**
***Published online 3 July 2023***

(*continued on overleaf*)

(*continued from overleaf*)

◆ *4710* The role of ventrolateral prefrontal cortex on voluntary emotion regulation of social pain
**Wenwen Yu, Yiwei Li, Xueying Cao, Licheng Mo, Yuming Chen, and Dandan Zhang**
***Published online 27 June 2023***

◆ *4722* Association of retinal nerve layers thickness and brain imaging in healthy young subjects from the i-Share-Bordeaux study
**Sara Cristina Lima Rebouças, Fabrice Crivello, Ami Tsuchida, Christophe Tzourio, Cédric Schweitzer, Jean-François Korobelnik, Cécile Delcourt, and Catherine Helmer**
***Published online 4 July 2023***

◆ *4738* Using predictive validity to compare associations between brain damage and behavior
**John F. Magnotti, Jaclyn S. Patterson, and Tatiana T. Schnur**
***Published online 7 July 2023***

◆ *4754* Multi-scale structural alterations of the thalamus and basal ganglia in focal epilepsy using 7T MRI
**Roy A. M. Haast, Benoit Testud, Julia Makhalova, Hugo Dary, Alexandre Cabane, Arnaud Le Troter, Jean-Philippe Ranjeva, Fabrice Bartolomei, and Maxime Guye**
***Published online 12 July 2023***

◆ *4772* Accounting for temporal variability in functional magnetic resonance imaging improves prediction of intelligence
**Yang Li, Xin Ma, Raj Sunderraman, Shihao Ji, and Suprateek Kundu**
***Published online 19 July 2023***

◆ *4792* Mapping tissue microstructure across the human brain on a clinical scanner with soma and neurite density image metrics
**Simona Schiavi, Marco Palombo, Domenico Zacà, Francesco Tazza, Caterina Lapucci, Lucio Castellan, Mauro Costagli, and Matilde Inglese**
***Published online 17 July 2023***

◆ *4812* Phonological and morphological literacy skills in English and Chinese: A cross-linguistic neuroimaging comparison of Chinese–English bilingual and monolingual English children
**Kehui Zhang, Xin Sun, Chi-Lin Yu, Rachel L. Eggleston, Rebecca A. Marks, Nia Nickerson, Valeria C. Caruso, Xiao-Su Hu, Twila Tardif, Tai-Li Chou, James R. Booth, and Ioulia Kovelman**
***Published online 23 July 2023***
